# Agomelatine prevents angiotensin II-induced endothelial and mononuclear cell adhesion

**DOI:** 10.18632/aging.203299

**Published:** 2021-07-22

**Authors:** Najiao Hong, Zhirong Ye, Yongjun Lin, Wensen Liu, Na Xu, Yan Wang

**Affiliations:** 1 Department of General Medicine, First Hospital of Quanzhou Affiliated to Fujian Medical University, Quanzhou 362000, Fujian, China; 2 Key Laboratory of Jilin Province for Zoonosis Prevention and Control, Institute of Military Veterinary Medicine, Academy of Military Medical Sciences, Changchun 130122, Jilin, China; 3 Department of Stomatology, Tibet Corps Hospital, Chinese People’s Armed Police Forces, Lhasa 850000, Tibet Autonomous Region, China

**Keywords:** angiotensin II, agomelatine, cardiovascular disease

## Abstract

Agomelatine is a non-selective melatonin receptor agonist and an atypical antidepressant with anti-inflammatory, neuroprotective, and cardioprotective effects. The renin-angiotensin system modulates blood pressure and vascular homeostasis. Angiotensin II (Ang II) and its receptor Ang II type I receptor (AT_1_R) are recognized as contributors to the pathogenesis of cardiovascular and cardiometabolic diseases, including diabetes, obesity, and atherosclerosis. The recruitment and attachment of monocytes to the vascular endothelium is a major event in the early stages of atherosclerosis and other cardiovascular diseases. In the present study, we demonstrate that agomelatine reduced Ang II-induced expression of AT_1_R while significantly inhibiting the attachment of monocytes to endothelial cells induced by Ang II and mediated by ICAM-1 and VCAM-1. Additionally, Ang II inhibited the expression of the chemokines CXCL1, MCP-1, and CCL5, which are critical in the process of immune cell recruitment and invasion. Agomelatine also suppressed the expression of TNF-α, IL-8, and IL-12, which are proinflammatory cytokines that promote endothelial dysfunction and atherogenesis. Importantly, we demonstrate that the inhibitory effect of agomelatine against the expression of adhesion molecules is mediated through the downregulation of Egr-1 signaling. Together, our findings provide evidence of a novel mechanism of agomelatine that may be practicable in the treatment and prevention of cardiovascular diseases.

## INTRODUCTION

The renin-angiotensin system (RAS) plays a central role in the pathophysiology of cardiovascular disease (CVD) due to its regulatory role in blood pressure, electrolyte balance, and cell growth. Angiotensin II (Ang II) is a significant component of the RAS that binds to the Ang II type I receptor (AT_1_R) to induce acute vasoconstriction, thereby increasing blood pressure [1]. Activation of AT_1_R by Ang II also induces vascular remodeling and endothelial dysfunction, which are major risk factors for atherosclerosis and other CVDs [2]. Endothelial cells are an essential cell type that lines the inner surface of blood and lymphatic vessels. The activation of endothelial cells by Ang II has been shown to contribute to pulmonary melanoma metastasis [3], microparticle formation [4], hypertension [5], and cerebral microvascular inflammation [6], among other things. Additionally, Ang II has been shown to cause endothelial cell apoptosis [7].

Ang II-induced endothelial dysfunction triggers the expression of vascular adhesion molecules, such as intracellular adhesion molecule-1 (ICAM-1) and vascular cellular adhesion molecule-1 (VCAM-1), and chemokines such as monocyte chemoattractant protein-1 (MCP-1) (also known as chemokine (C-C motif) ligand 2 (CCL2)), chemokine (C-X-C motif) ligand 1 (CXCL1), and C-C motif chemokine ligand 5 (CCL5) [8, 9]. In atherosclerosis, dysfunctional endothelial cells release MCP-1 to recruit immune cells to adhere to and invade the arterial wall, where they accumulate along with lipids and lipoproteins, thereby forming atherosclerotic lesions [10]. CXCL1 and CCL5 promote monocyte and leukocyte accumulation in atherosclerotic lesions as well as monocyte arrest on the endothelium [11, 12]. Inhibiting the recruitment and adhesion of immune cells is a widely explored treatment approach for atherosclerosis and other cardiometabolic diseases.

Endothelial inflammation and hypertension induced by Ang II are implicated in the pathogenesis of CVDs, especially atherosclerosis. Inhibiting the activity of the RAS and Ang II is regarded as a valuable therapeutic option to halt the progression of atherosclerosis as it can reduce inflammation, oxidative stress, plaque burden, and improve arterial wall thickness [13]. Ang II-induced inflammation has also been shown to contribute to cardiac remodeling and fibrosis [14]. Tumor necrosis factor-α (TNF-α) is well recognized as a central mediator of inflammation in numerous diseases. Ang II-induced expression of TNF-α causes endothelial cells to take on a pathological phenotype and leads to the production of matrix metalloproteinase-2 (MMP-2), which is associated with increased plaque vulnerability [15]. Interleukin (IL)-8 and IL-12 expression is also induced by Ang II. IL-8 mediates the expression of adhesion molecules, including ICAM-1, thereby modulating monocyte adhesiveness [16, 17]. Meanwhile, increased expression of IL-12 is associated with atherosclerosis, coronary artery disease, atrial fibrillation, aortic dissection, cardiomyopathy, and viral myocarditis [18, 19]. Early growth response-1 (Egr-1) is a zinc finger transcription factor and an immediate early gene recognized as a master regulator of inflammation in CVD. Egr-1 is abundantly expressed in atherosclerotic lesions and promotes the expression of inflammatory cytokines and cellular adhesion molecules by macrophages [20]. Previous research has shown that Ang II can induce Egr-1 expression through the AT_1_R/c-Jun N terminal kinase (JNK)/extracellular signal-regulated kinase (ERK) signaling pathway [21]. Inhibiting the expression of Egr-1 is viewed as a potential treatment strategy for atherosclerosis.

Agomelatine is an atypical antidepressant and a non-selective MT_1_/MT_2_ melatonin receptor agonist that has been shown to exert chronotropic [22], neuroprotective [23], and anti-neuropathic pain effects [24]. Pharmacodynamics studies indicate that Agomelatine could be effective in treating major depressive disorder (MDD) and other non-typical depression syndromes such as bipolar depression, anxiety disorders, alcohol dependence, migraines, mood, and anxiety disorders [25–27]. 80% Agomelatine could be absorbed after oral administration. However, its bioavailability is lower than 5%. 90% agomelatine is reported to be metabolized by cytochrome P450 (CYP) 1A2 and the left 10% is metabolized by CYP 450 2C9 isoforms.

Additionally, agomelatine has demonstrated cardioprotective effects by inhibiting the activation of inflammatory nuclear factor-κB (NF-κB) signaling [28], stimulating melatonin receptors [29], and preventing doxorubicin-induced cardiotoxicity and ECG abnormalities [30]. Interestingly, recent research has demonstrated a hypotensive effect of agomelatine in rat models of intraocular hypertension and glaucoma patients [31, 32]. However, it is unclear whether agomelatine interacts with the RAS to alleviate CVD. Agomelatine has also demonstrated anti-inflammatory effects. For example, agomelatine reduced the inflammatory response induced by lipopolysaccharide in rats via suppression of IL-1β and IL-6 [33, 34]. In the present study, we assessed the potential of agomelatine to protect against Ang II-induced endothelial dysfunction by measuring its effect on Egr-1-mediated monocyte adhesion to endothelial cells and the expression of inflammatory cytokines and chemokines.

## MATERIALS AND METHODS

### Cell culture and treatment

Human umbilical vascular endothelial cells (HUVECs) (Lonza, Basel, Switzerland) and human monocytic leukemia cell line THP-1 cells (ATCC, Manassas, USA) were cultured in EBM-2 medium with 10% heat-inactivated FBS, L-glutamine, and antibiotics (penicillin/streptomycin) (Life Technologies, USA) as previously described [32, 33]. The cells were incubated overnight in a 5% CO_2_ incubator at a temperature of 37° C. HUVECs were exposed to Ang II (1 μM) with or without agomelatine (10, 20 μM) for 24 h.

### Real-time PCR

Qiazol reagent (Qiagen, USA) was used following the manufacturer's instructions to extract the total RNA from HUVECs. The isolated RNA was treated with DNase I for 2 h at 37° C. Then, cDNA was synthesized using a high-capacity cDNA synthesis kit (Applied Biosystems, USA) and a reverse transcriptional PCR (RT-PCR) kit in accordance with the manufacturers' instructions. For real-time polymerase chain reaction (PCR) analysis, 2 μl cDNA was analyzed on an Applied Biosystems 7500 Real-Time PCR System using the SYBR Green PCR Master Mix method to determine the expression levels of the target genes. The results were analyzed using the 2^-ΔΔCt^ method and were presented as normalized to the expression level of the housekeeping gene GAPDH. The following primers were used: VCAM-1: 5'-CTTAAAATGCCTGGGAAGATGGT-3’ (forward); 5’-GTCAATGAGACGGAGTCACCAAT-3’ (reverse); ICAM-1: 5’-CGATGACCATCTACAGCTTTCCGG-3’ (forward); 5’-GCTGCTACCACAGTGATGATGACAA-3 (reverse); TNF-α: 5’-GTCACTCATTGCTGAGCCTCT-3’ (forward); 5’-AGCTTCTTCCCACCCACAAG-3’ (reverse); IL-8: 5’-TTTCTGTTAAATCTGGCAACCCTAGT-3’ (forward); 5’-ATAAAGGAGAAACCAAGGCACAGT-3’ (reverse); GAPDH (internal control), 5’-GGAGAAGGCTGGGGCTCAT-3’ (forward) and 5’-TGATGGCATGGACTGTGGTC-3’ (reverse).

### Western blot analysis

Total intracellular protein was isolated using cell lysis buffer supplemented with protease and phosphatase inhibitor cocktail (Sigma-Aldrich, USA). Then, 10% sodium dodecyl sulfate-polyacrylamide gel electrophoresis (SDS-PAGE) was used to separate the isolated proteins, which were then transferred onto polyvinylidene fluoride (PVDF) membranes. The membranes were blocked with 5% non-fat milk in TBST to block the non-specific sites. The membranes were then incubated with primary antibodies overnight at 4° C, washed 3 times in PBS buffer, and incubated with horseradish peroxidase (HRP)-conjugated secondary antibody at RT for 2 h. The bands on the blots were visualized using Pierce ECL western blotting substrate [35]. The following antibodies were used in this study: β-actin (#4970, Cell Signaling Technology, USA), Egr-1 (#ab194357, Abcam, USA), AT1R (#ab124734, Abcam, USA), and anti-rabbit IgG, HRP-linked secondary antibody (#7074, Cell Signaling Technology, USA).

### ELISA

The concentrations of proteins secreted into the supernatant were assessed using commercial ELISA kits from R&D Systems. The following ELISA kits were used: CXCL1 (#DGR00), MCP-1 (#DCP00), CCL-5 (#DRN00B), VCAM-1 (DVC00), ICAM-1 (DCD540), TNF-α (DTA00D), IL-12(D1200), and IL-8(D8000C). The assay process was performed following the manufacturer's instructions. Data collection was performed using a 96-well plate reader spectrometry. A standardized 4-PL curve was used to obtain absolute values, and the relative levels of the target proteins are presented as normalized to the total protein amounts.

### Calcein-AM

For the cellular adhesion experiment, 5 × 10^5^ THP-1 monocytes stained with the cell-permanent dye calcein-AM with green fluorescence were co-cultured with 1 × 10^5^ confluent HUVECs for 2 h. After that, unbounded THP-1 cells were washed away by using PBS. Fluorescent signals were visualized using a fluorescent microscope. The number of attached monocytes was quantified using Image J software.

### Egr-1 overexpression

The construct of Egr-1 expression (pCDNA3.1-Egr-1) was obtained from a commercial source (Addgene, USA). To overexpress Egr-1 gene in HUVECs, 2 μg of purified plasmid was transfected into HUVECs growing on 60-mm culture dishes by Lipofectamine LTX Reagent (Thermo Fisher Scientific Inc, USA). The expression level of Egr-1 was verified by RT-PCR and Western blot experiments.

### Statistics

The experimental results are expressed as means ± standard error of the mean (S.E.M.). One-way analysis of variance (ANOVA) followed by Bonferroni’s post-hoc test was used to obtain statistical values for multi-group comparisons. A P-value of < 0.05 was considered statistically significant [36].

## RESULTS

### The expression of the Ang II receptor AT_1_R

**Figure 1:**
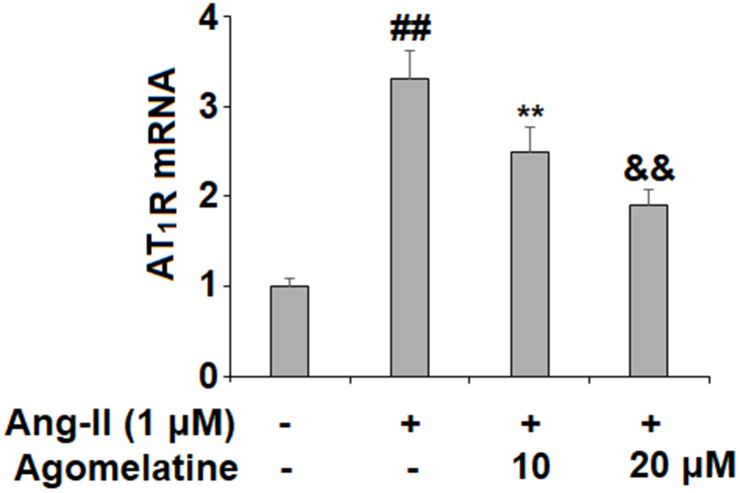
**Agomelatine reduced Ang II-induced the expression of Ang II type 1 receptor (AT_1_ receptor) in HUVECs.** HUVECs were treated with Ang II (1 μM) with or without agomelatine (10, 20 μM) for 24 h. mRNA of AT_1_ receptor (##, **, &&, P<0.01 vs. vehicle control, Ang II, Ang II+ 10 μM agomelatine group).

To investigate the effect of Agomelatine in vascular cells, we added Agomelatine in Ang II stimulated endothelial cells. Ang II activates endothelial cells by binding to its type I receptor AT_1_R on the endothelial surface to induce oxidative stress, endothelial dysfunction, and apoptosis [37, 38]. We hypothesized that the presence of Agomelatine could affect the cellular stress by Ang II. Here, we found that the introduction of 10 and 20 μM agomelatine prevented the increase in AT_1_R expression induced by exposure to Ang II, with the higher dose having a more robust inhibitory effect (Figure 1).

### Adhesion of monocytes to endothelial cells

**Figure 2:**
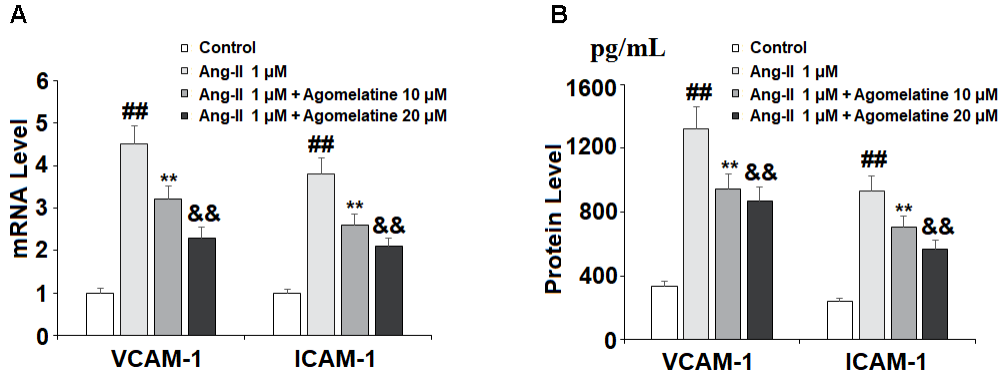
**The effects of agomelatine in Ang II-induced expression of VCAM-1 and ICAM-1 in HUVECs.** HUVECs were treated with Ang II (1 μM) with or without agomelatine (10, 20 μM) for 24 h. (**A**) mRNA levels of VCAM-1 and ICAM-1; (**B**) Protein of VCAM-1 and ICAM-1 (##, **, &&, P<0.01 vs. vehicle control, Ang II, Ang II+ 10 μM agomelatine group).

**Figure 3:**
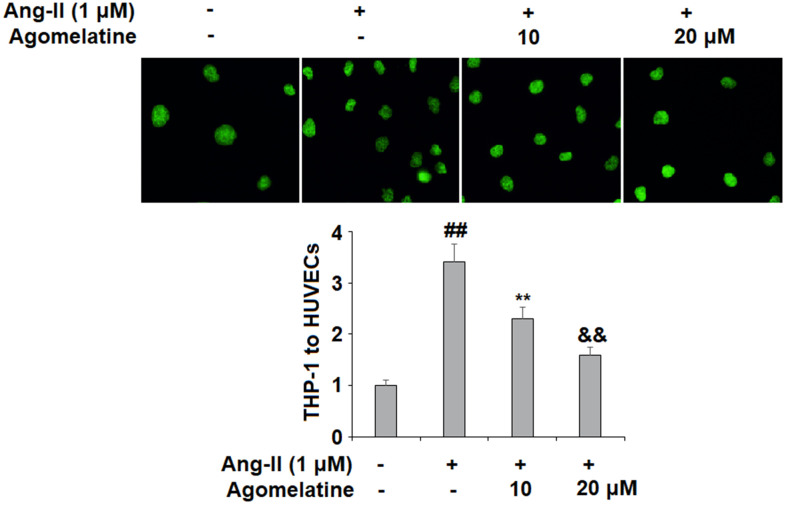
**The effects of agomelatine in Ang II-induced attachment of THP-1 monocytes to HUVECs.** HUVECs were treated with Ang II (1 μM) with or without agomelatine (10, 20 μM) for 24 h. Calcein-AM staining method was used to measure attachment of THP-1 cells to HUVECs (##, **, &&, P<0.01 vs. vehicle control, Ang II, Ang II+ 10 μM agomelatine group).

Next, we investigated the effect of agomelatine on Ang II-induced secretion of adhesion molecules and subsequent adhesion of THP-1 monocytes to HUVECs. Real-time PCR and ELISA were used to determine the mRNA and protein levels of VCAM-1 and ICAM-1, two key adhesion molecules. As shown in Figure 2A, 2B, 10 and 20 μM agomelatine dose-responsively inhibited the increase in the mRNA and protein expression of VCAM-1 and ICAM-1 induced by Ang II, respectively. We then performed an adhesion assay experiment to test whether the inhibition of these adhesion molecules by agomelatine results in reduced monocyte adhesion to endothelial cells. Indeed, the results of calcein-AM staining in Figure 3 indicate that agomelatine reduced the number of adhered monocytes in a dose-responsive manner.

### The expression of proinflammatory chemokines

**Figure 4:**
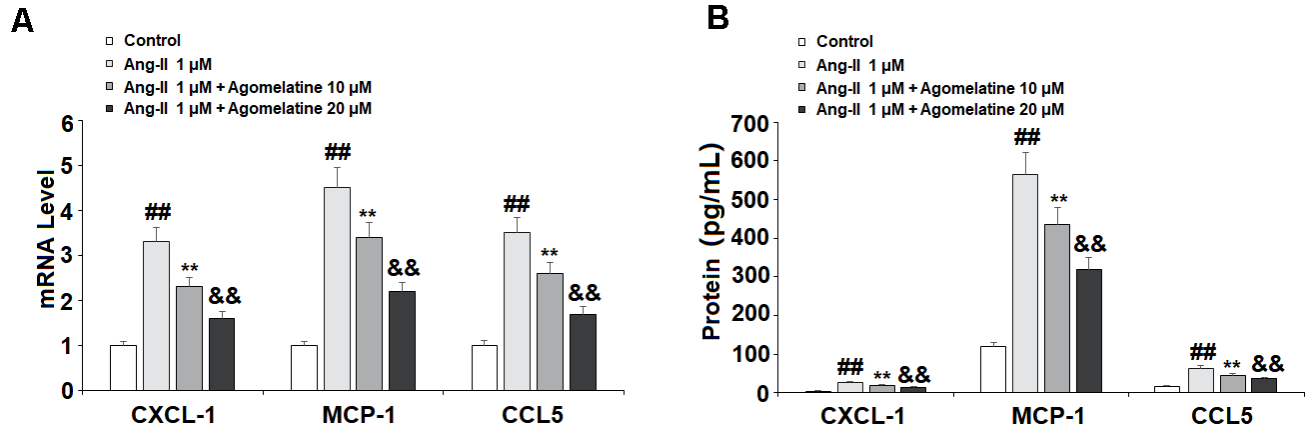
**The effects of agomelatine in Ang II-induced chemokine production in HUVECs.** HUVECs were treated with Ang II (1 μM) with or without agomelatine (10, 20 μM) for 24 h. (**A**) mRNA of CXCL1, MCP-1, and CCL5; (**B**) Protein of CXCL1, MCP-1, and CCL5 (##, **, &&, P<0.01 vs. vehicle control, Ang II, Ang II+ 10 μM agomelatine group).

Ang II induces the expression of proinflammatory cytokines and chemokines in endothelial cells. We investigated the effect of agomelatine on the expression of proinflammatory chemokines, including CXCL1, MCP-1, and CCL5. These three chemokines play a major role in the pathogenesis of CVDs by recruiting immune cells to invade the endothelium. The results of real-time PCR and ELISA show that while Ang II significantly increased the mRNA (Figure 4A) and protein (Figure 4B) expression of these chemokines, the two doses of agomelatine ameliorated this effect by roughly half.

### The expression of proinflammatory cytokines

**Figure 5:**
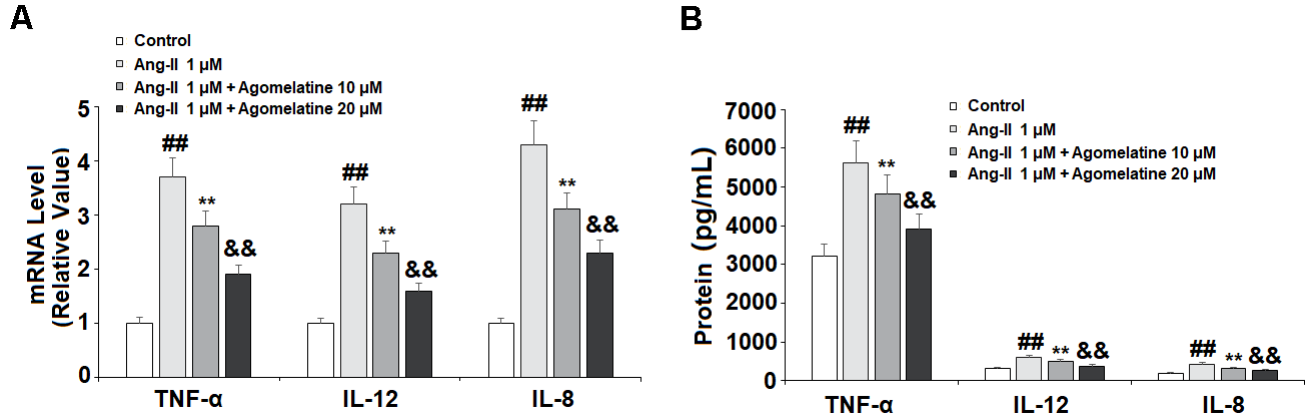
**The effects of agomelatine in Ang II-induced proinflammatory cytokine production in HUVECs.** HUVECs were treated with Ang II (1 μM) with or without agomelatine (10, 20 μM) for 24 h. (**A**) mRNA of TNF-α, IL-12, and IL-8; (**B**) Protein of TNF-α, IL-12, and IL-8 (##, **, &&, P<0.01 vs. vehicle control, Ang II, Ang II+ 10 μM agomelatine group).

The activated endothelial cells produce high levels of proinflammatory cytokines, which play a critical role in the pathogenesis of CVDs. Therefore, we measured the expression levels of TNF-α, IL-8, and IL-12 in Ang II-challenged HUVECs. As shown in Figure 5A, 5B, Ang II significantly increased the expression of these three cytokines at both the mRNA and protein levels. At the same time, 10 and 20 μM agomelatine dose-responsively suppressed this increase in cytokine expression.

### Involvement of Egr-1 signaling

**Figure 6:**
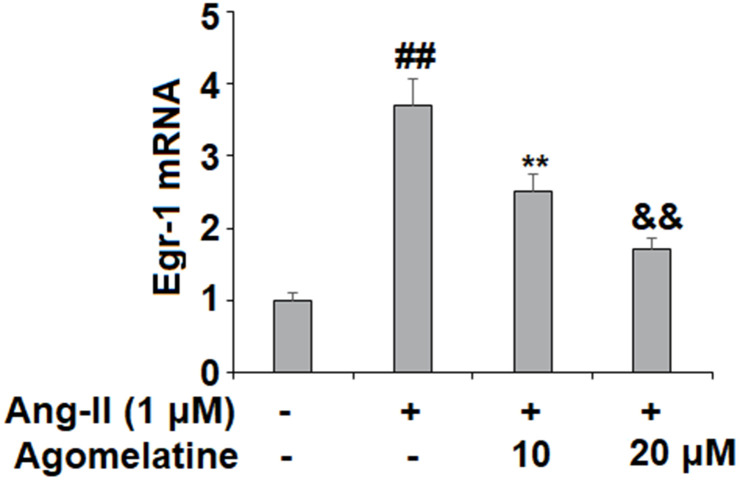
**Agomelatine reduced Ang II-induced expression of Egr-1 in HUVECs.** HUVECs were treated with Ang II (1 μM) with or without agomelatine (10, 20 μM) for 24 h. mRNA of Egr-1 (##, **, &&, P<0.01 vs. vehicle control, Ang II, Ang II+ 10 μM agomelatine group).

**Figure 7:**
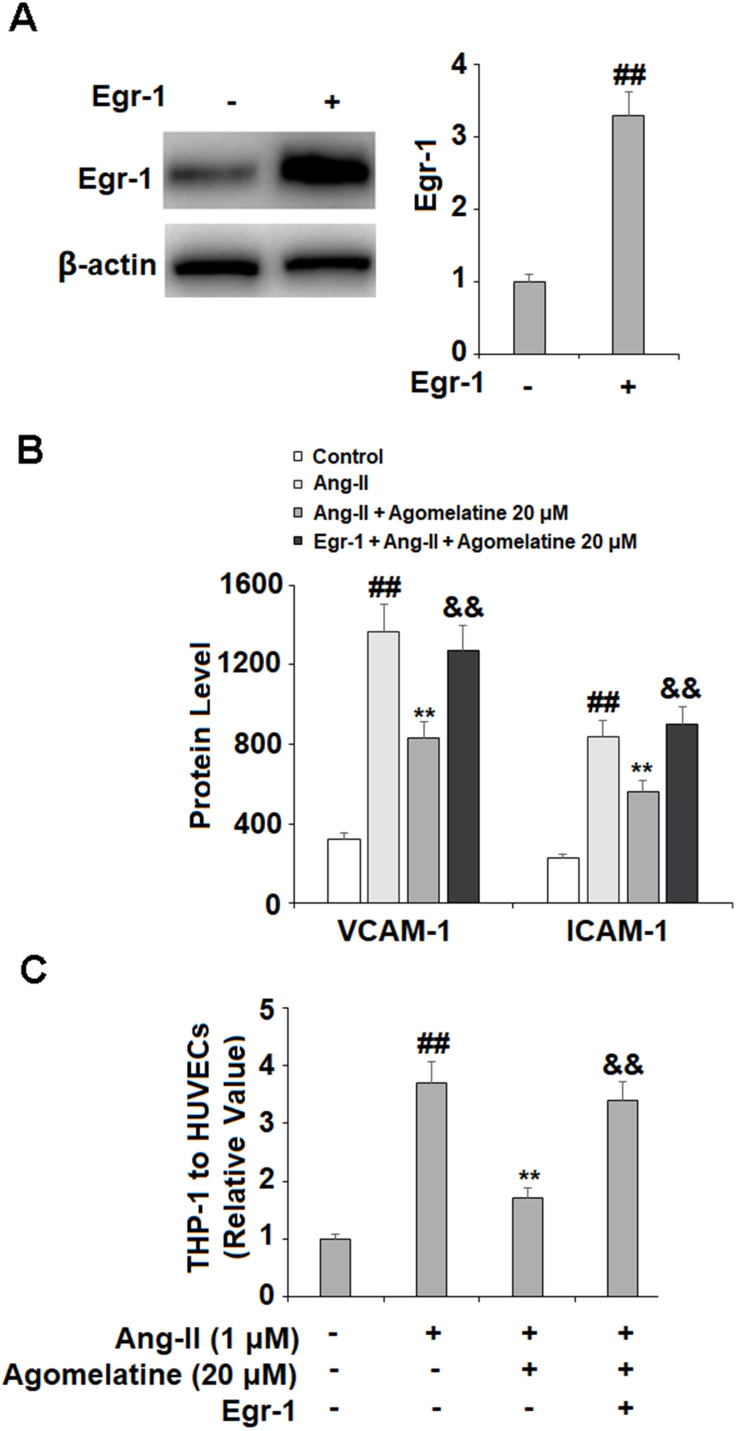
**Overexpression of Egr-1 abolished the protective effect of agomelatine against Ang II in HUVECs.** HUVECs were transfected with Egr-1 plasmid, followed by stimulation with Ang II (1 μM) with or without agomelatine (20 μM) for 24 h. (**A**) Successful overexpression of Egr-1; (**B**) Protein of VCAM-1 and ICAM-1; (**C**) Calcein-AM staining method was used to measure attachment of THP-1 cells to HUVECs (##, **, &&, P<0.01 vs. vehicle control, Ang II, Ang II+ 20 μM agomelatine group).

Finally, we set out to determine whether Egr-1 signaling was involved in mediating the inhibitory effects of agomelatine against monocyte-endothelial cell adhesion described above. The results in Figure 6 reveal that while Ang II induced a significant increase in Egr-1, agomelatine dose-responsively ameliorated this effect. To confirm whether this reduction in Egr-1 signaling is involved in the mechanism of agomelatine, we performed an Egr-1 overexpression experiment. The results in Figure 7A–7C demonstrate that overexpression of Egr-1 abolished the activity of agomelatine on the expression of VCAM-1 and ICAM-1 as well as the attachment of monocytes to endothelial cells. Thus, agomelatine inhibits the attachment of monocytes to endothelial cells by reducing adhesion molecule expression through the inhibition of Egr-1.

## DISCUSSION

Agomelatine is an atypical antidepressant that displays dual action by acting as an agonist of the MT_1_ and MT_2_ melatonergic receptors and an antagonist of 5-hydroxytryptamine receptor 2C (5-HTR2C). Agomelatine is used for the treatment of depressive disorders including bipolar depression [39], anxiety [40], and seasonal affective disorder [41]. As a new medication, only a few studies investigated the effects of agomelatine in various pathological conditions, such as CVD and atherosclerosis. Recent study reports that agomelatine possesses certain cardioprotective benefits, and it has a protective role against ischemia-reperfusion injury and myocardial infarction [42]. Clinical study suggests that agomelatine is safe in patients with CVD [43]. As a melatonin receptor agonist, agomelatine and melatonin share some common mechanisms. For example, melatonin has been shown to confer similar cardioprotective effects against ischemia-reperfusion injury and myocardial infarction [44, 45].

Interestingly, both melatonin and agomelatine can modulate impaired circadian rhythm through their chronotropic effects [22, 46], which has been linked with aggravated atherogenesis [47]. Melatonin has also been shown to hinder the progression of atherosclerosis by mediating NLRP3 inflammasome activation and SIRT3 activity [46]. Therefore, the investigation of the underlying mechanism that agomelatine is involved could have potential implications in CVD diseases. In the present study, we provide evidence that agomelatine has an anti-atherogenic effect by ameliorating vascular inflammation and monocyte adhesion via inhibiting the Egr-1 signaling.

Activation of AT_1_R by Ang II has been linked with numerous pathologies. In cardiometabolic diseases, including diabetes, obesity, and hypertension, agonism of AT_1_R induces an inflammatory cascade, oxidative stress, and increased expression of cellular adhesion molecules by endothelial cells. This process plays a key role in endothelial dysfunction in the early stages of atherosclerosis [46]. Here, we found that agomelatine inhibited AT_1_R activation by Ang II, which was accompanied by reduced expression of adhesion molecules, cytokines, and chemokines. It has been consistently shown that Ang II stimulation increased its receptor AT1R in neuronal cells via NF-κB and other regulators [47, 48]. Since hyperactivation of AT1R is detrimental to vascular function [2], the alleviation of Agomelatine on AT1R indicates it has potential protection on vascular endothelial cells. Next, we assessed the action of agomelatine on vascular adhesion molecules. Agomelatine significantly reduced the expression of VCAM-1 and ICAM-1, which has not been reported before. Consistently, both melatonin and inhibition of AT_1_R have been shown to inhibit increased expression of ICAM-1 and VCAM-1 [49–51]. We further demonstrated that agomelatine significantly reduced the adhesion of monocytes to endothelial cells, which is likely mediated via reduced expression of adhesion molecules and chemokines. Here, we found that agomelatine treatment could reduce the expression of the chemokines CXCL1, MCP-1, and CCL5. CXCL1 and its receptor CXCR2 have been shown to mediate Ang II-induced cardiac remodeling by recruiting monocytes to infiltrate the heart [52].

Meanwhile, a recent study showing that melatonin could reduce MCP-1 expression suggested that agomelatine may share this effect, which the present study has determine to be correct [53]. CCL5 may act as a regulatory factor against Ang II-induced hypertension [54], but has also been recognized as an indicator of short-term mortality in patients with CVD [55]. The inhibitory role of agomelatine on these chemokines indicates that it could alleviate the recruitment of immune cells to the surface of endothelial cells. Indeed, our adhesion experiment showed that agomelatine suppressed the amounts of THP-1 cells adhesion to HUVECs. We have to address the limitation of using THP-1 cells in this experiment. Although THP-1 cells have been widely used as a cell line for the physiological mechanism of monocytes and macrophages in the cardiovascular system [56]. However, due to its derivation of acute monocytic leukemia, the definitive conclusion of using THP-1 cells to mimic monocytes and macrophages in the vasculature requires further investigation by using primary cells or *in vivo* models.

Furthermore, we demonstrated an anti-inflammatory effect of agomelatine mediated by reduced expression of TNF-α, IL-8, and IL-12. The role of these proinflammatory cytokines in CVDs has been well-studied. The expression of these cytokines has been recognized as a common pathological mechanism between depression and CVD [57]. Concordant with our findings, previous research has reported a robust anti-inflammatory effect of Agomelatine [30]. Another study suggests that the effectiveness and severity of Agomelatine in the treatment of depression are associated with circulating CRP levels in depression patients. Moreover, a higher CRP level is associated with more depressive symptoms at baseline [58]. Additionally, agomelatine may increase serum neurotrophin levels such as BNDF [59]. These facts suggest that Agomelatine could possess a wide range of modulation on cardiovascular cells and neuronal cells.

Egr-1 is recognized as a key mediator of Ang II-induced intimal hyperplasia, monocyte adhesion, and atherosclerosis [60]. Increased expression of Egr-1 is also associated with endothelial inflammation, exacerbated oxygen-glucose deprivation/reperfusion-induced injury, and impaired diabetic wound healing capacity [61–63]. Ang II induces the expression of Egr-1 through AT_1_R/JNK and ERK signaling [64]. The interaction between Egr-1 and agomelatine has not yet been clarified. Melatonin has been shown to downregulate Egr-1 in the pars tuberalis in sheep, thereby influencing circadian gene expression [65]. Here, we demonstrate that agomelatine treatment inhibits Ang II-induced Egr-1 expression. Our findings further demonstrate that the effects of agomelatine against monocyte adhesion to endothelial cells were mediated through Egr-1, as overexpression of Egr-1 abolished the inhibitory effect of agomelatine on adhesion molecules, which was confirmed through a cellular adhesion assay.

Taken together, our findings demonstrate the potential of agomelatine, a unique melatonin analog, to hinder the development of atherosclerosis and other CVDs or cardiometabolic disorders. Agomelatine significantly inhibited the expression of AT_1_R induced by Ang II, as well as the expression of several critical proinflammatory cytokines including TNF-α, IL-8, and IL-12. Additionally, agomelatine exerted a robust inhibitory effect against Egr-1-mediated attachment of monocytes to endothelial cells via downregulation of ICAM-1 and VCAM-1. As this study has certain limitations, such as the use of an *in vitro* model and further research using ideal animal models and exploring the mechanism of agomelatine in greater depth is warranted.
